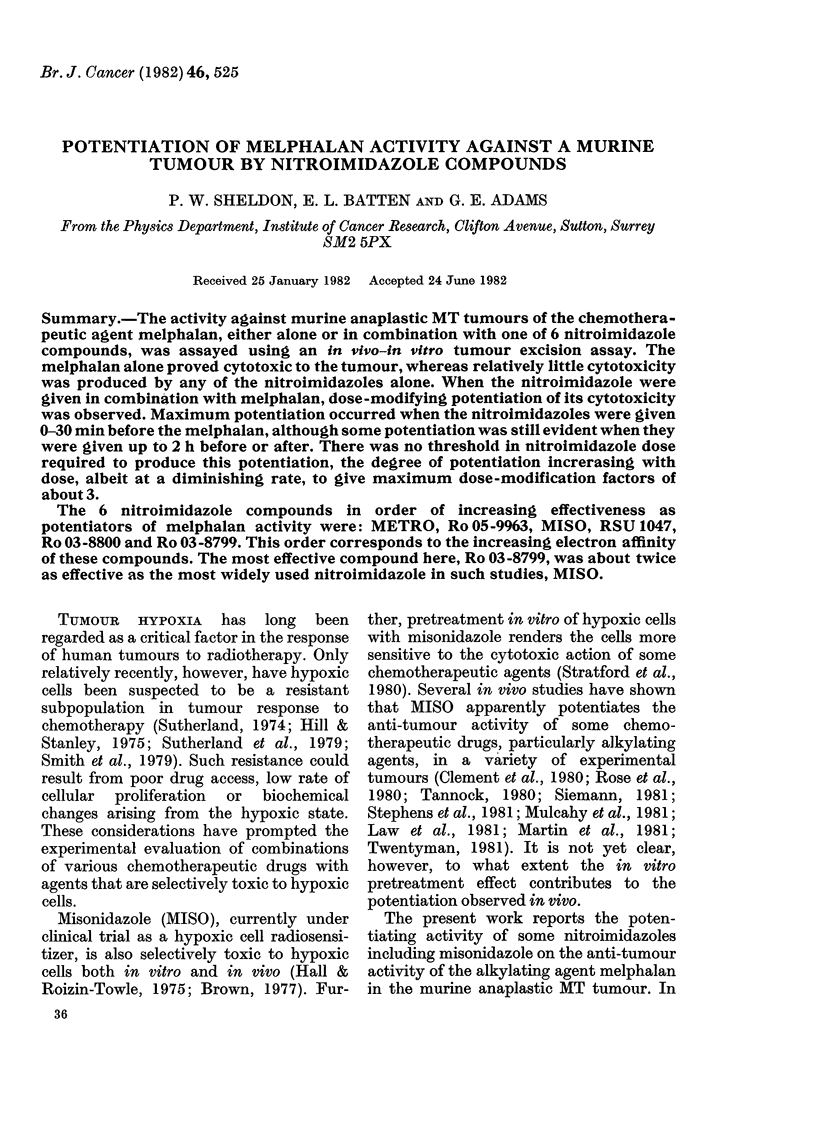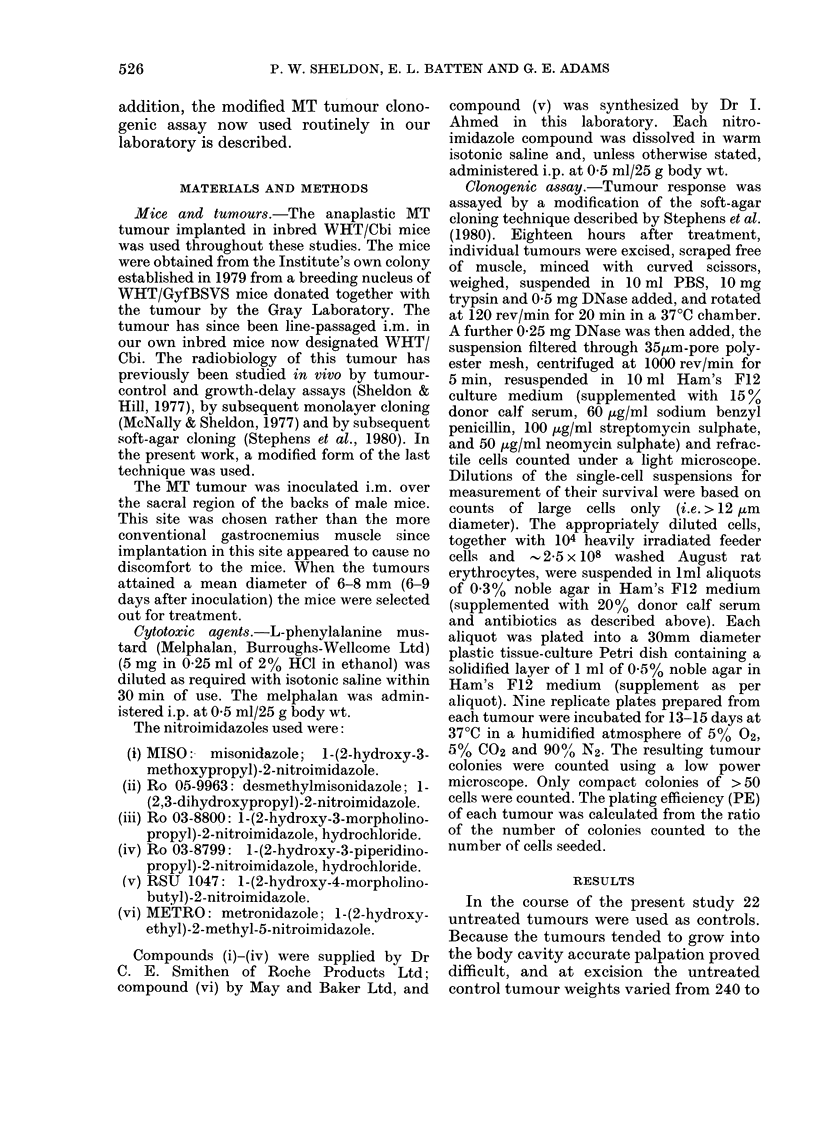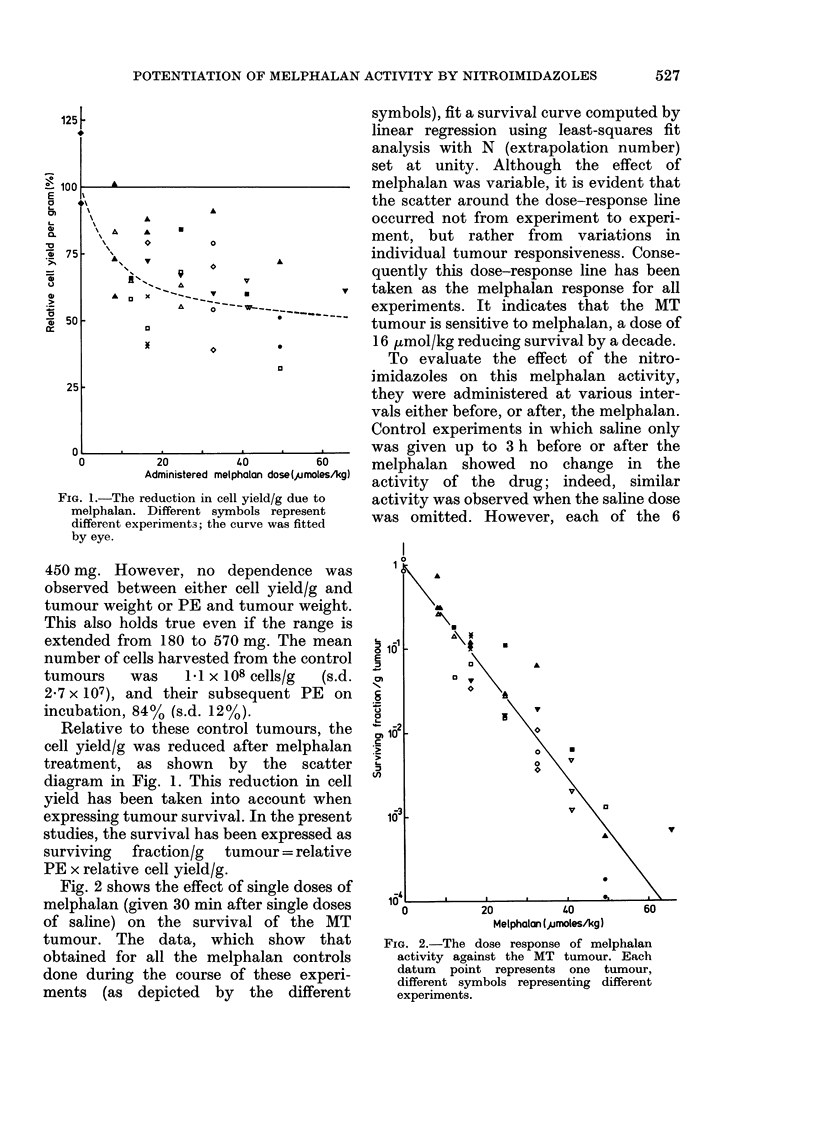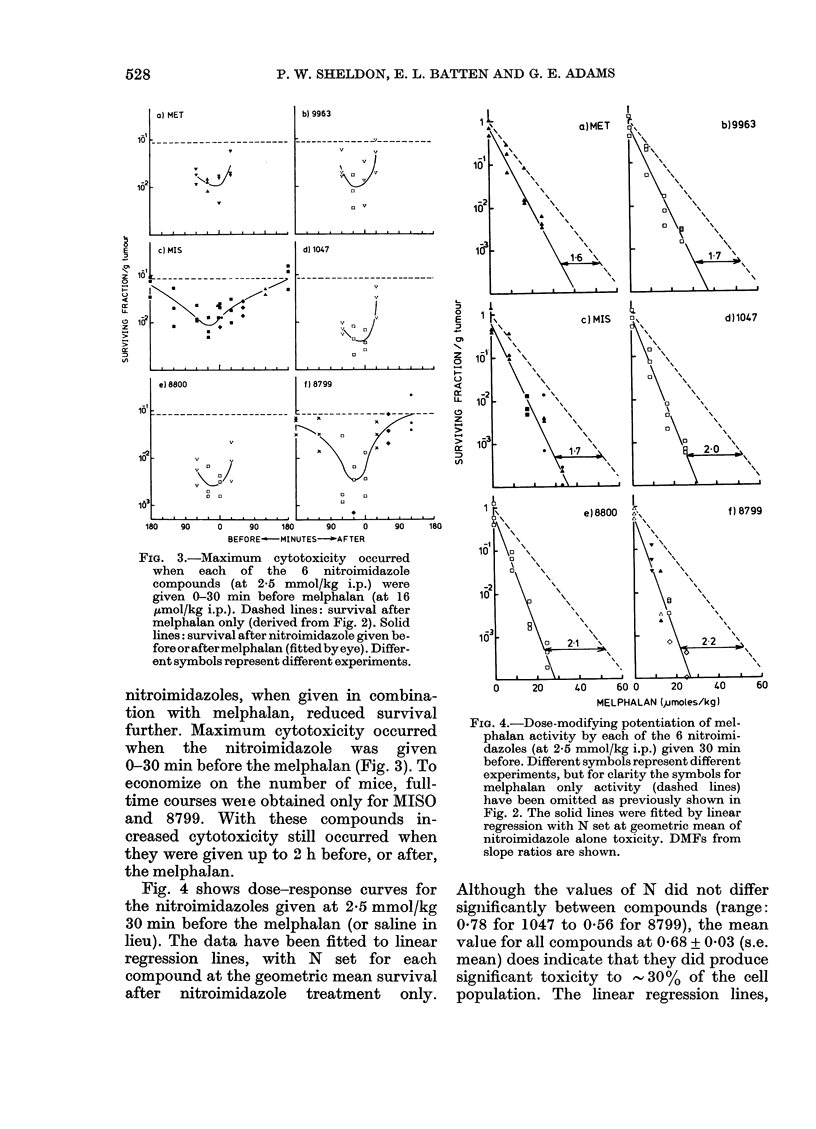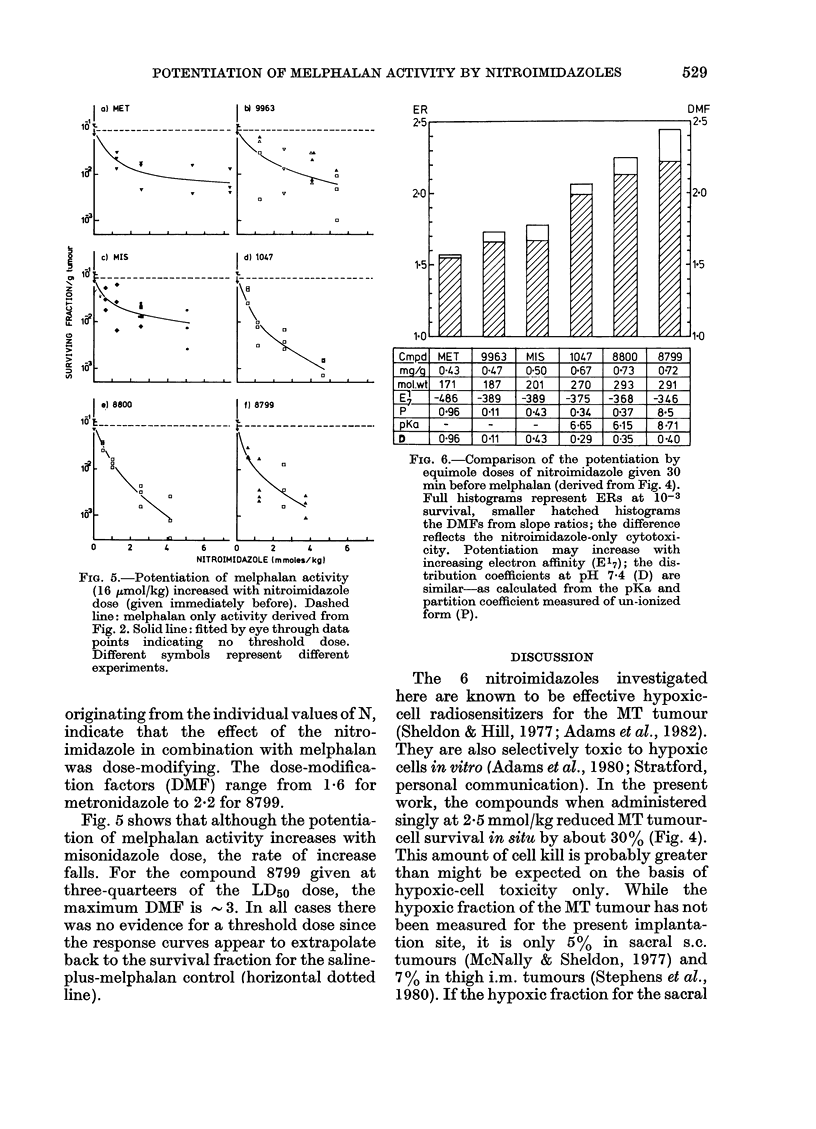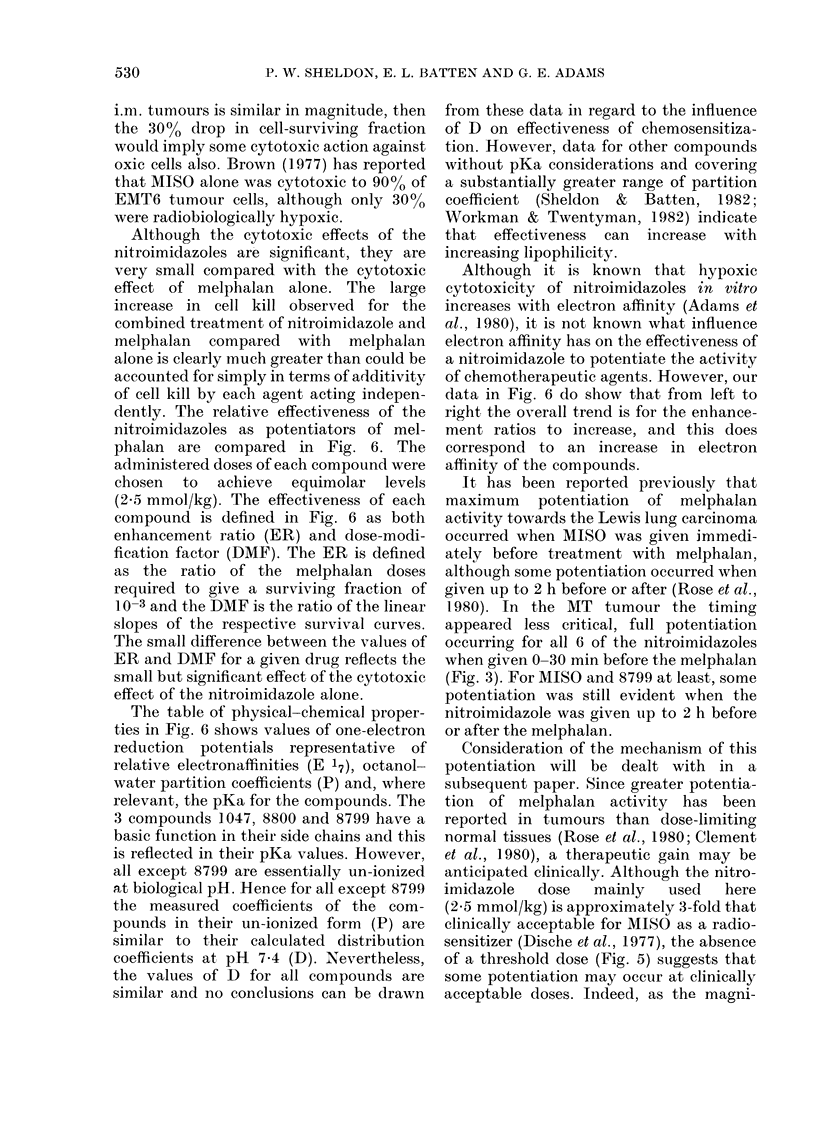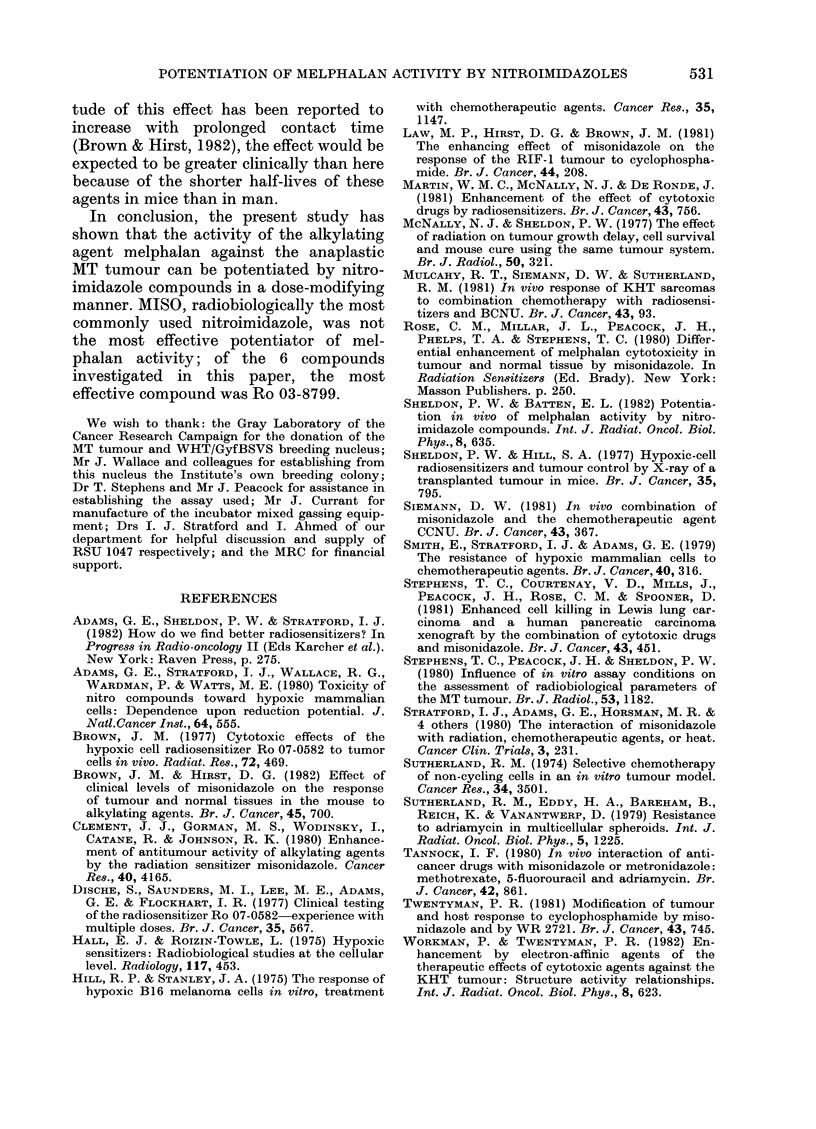# Potentiation of melphalan activity against a murine tumour by nitroimidazole compounds.

**DOI:** 10.1038/bjc.1982.236

**Published:** 1982-10

**Authors:** P. W. Sheldon, E. L. Batten, G. E. Adams

## Abstract

The activity against murine anaplastic MT tumours of the chemotherapeutic agent melphalan, either alone or in combination with one of 6 nitroimidazole compounds, was assayed using an in vivo-in vitro tumour excision assay. The melphalan alone proved cytotoxic to the tumour, whereas relatively little cytotoxicity was produced by any of the nitroimidazoles alone. When the nitroimidazole were given in combination with melphalan, dose-modifying potentiation of its cytotoxicity was observed. Maximum potentiation occurred when the nitroimidazoles were given 0--30 min before the melphalan, although some potentiation was still evident when they were given up to 2 h before or after. There was no threshold in nitroimidazole dose required to produce this potentiation, the degree of potentiation increasing with dose, albeit at a diminishing rate, to give maximum dose-modification factors of about 3. The 6 nitroimidazole compounds in order of increasing effectiveness as potentiators of melphalan activity were: METRO, Ro 05-9963, MISO, RSU 1047, Ro 03-8800 and Ro 03-8799. This order corresponds to the increasing electron affinity of these compounds. The most effective compound here, Ro 03-8799, was about twice as effective as the most widely used nitroimidazole in such studies, MISO.


*Br. J. Cancer* (1982) **46**, 525

### POTENTIATION OF MELPHALAN ACTIVITY AGAINST A MURINE TUMOUR BY NITROIMIDAZOLE COMPOUNDS

P. W. SHELDON, E. L. BATTEN AND G. E. ADAMS

*From the Physics Department, Institute of Cancer Research, Clifton Avenue, Sutton, Surrey SM2 5PX*

Received 25 January 1982 Accepted 24 June 1982

**Summary.—The activity against murine anaplastic MT tumours of the chemotherapeutic agent melphalan, either alone or in combination with one of 6 nitroimidazole compounds, was assayed using an *in vivo-in vitro* tumour excision assay. The melphalan alone proved cytotoxic to the tumour, whereas relatively little cytotoxicity was produced by any of the nitroimidazoles alone. When the nitroimidazole were given in combination with melphalan, dose-modifying potentiation of its cytotoxicity was observed. Maximum potentiation occurred when the nitroimidazoles were given 0–30 min before the melphalan, although some potentiation was still evident when they were given up to 2 h before or after. There was no threshold in nitroimidazole dose required to produce this potentiation, the degree of potentiation incrementing with dose, albeit at a diminishing rate, to give maximum dose-modification factors of about 3.**

**The 6 nitroimidazole compounds in order of increasing effectiveness as potentiators of melphalan activity were: METRO, Ro 05-9963, MISO, RSU 1047, Ro 03-8800 and Ro 03-8799. This order corresponds to the increasing electron affinity of these compounds. The most effective compound here, Ro 03-8799, was about twice as effective as the most widely used nitroimidazole in such studies, MISO.**

TUMOUR HYPOXIA has long been regarded as a critical factor in the response of human tumours to radiotherapy. Only relatively recently, however, have hypoxic cells been suspected to be a resistant subpopulation in tumour response to chemotherapy (Sutherland, 1974; Hill & Stanley, 1975; Sutherland *et al.*, 1979; Smith *et al.*, 1979). Such resistance could result from poor drug access, low rate of cellular proliferation or biochemical changes arising from the hypoxic state. These considerations have prompted the experimental evaluation of combinations of various chemotherapeutic drugs with agents that are selectively toxic to hypoxic cells.

Misonidazole (MISO), currently under clinical trial as a hypoxic cell radiosensitizer, is also selectively toxic to hypoxic cells both *in vitro* and *in vivo* (Hall & Roizin-Towle, 1975; Brown, 1977). Further, pretreatment *in vitro* of hypoxic cells with misonidazole renders the cells more sensitive to the cytotoxic action of some chemotherapeutic agents (Stratford *et al.*, 1980). Several *in vivo* studies have shown that MISO apparently potentiates the anti-tumour activity of some chemotherapeutic drugs, particularly alkylating agents, in a variety of experimental tumours (Clement *et al.*, 1980; Rose *et al.*, 1980; Tannock, 1980; Siemann, 1981; Stephens *et al.*, 1981; Mulcahy *et al.*, 1981; Law *et al.*, 1981; Martin *et al.*, 1981; Twentyman, 1981). It is not yet clear, however, to what extent the *in vitro* pretreatment effect contributes to the potentiation observed *in vivo*.

The present work reports the potentiating activity of some nitroimidazoles including misonidazole on the anti-tumour activity of the alkylating agent melphalan in the murine anaplastic MT tumour. In addition, the modified MT tumour clonogenic assay now used routinely in our laboratory is described.

### MATERIALS AND METHODS

*Mice and tumours.*—The anaplastic MT tumour implanted in inbred WHT/Cbi mice was used throughout these studies. The mice were obtained from the Institute's own colony established in 1979 from a breeding nucleus of WHT/GyfBSVS mice donated together with the tumour by the Gray Laboratory. The tumour has since been line-passaged i.m. in our own inbred mice now designated WHT/Cbi. The radiobiology of this tumour has previously been studied *in vivo* by tumour-control and growth-delay assays (Sheldon & Hill, 1977), by subsequent monolayer cloning (McNally & Sheldon, 1977) and by subsequent soft-agar cloning (Stephens *et al.*, 1980). In the present work, a modified form of the last technique was used.

The MT tumour was inoculated i.m. over the sacral region of the backs of male mice. This site was chosen rather than the more conventional gastrocnemius muscle since implantation in this site appeared to cause no discomfort to the mice. When the tumours attained a mean diameter of 6–8 mm (6–9 days after inoculation) the mice were selected out for treatment.

*Cytotoxic agents.*—L-phenylalanine mustard (Melphalan, Burroughs-Wellcome Ltd) (5 mg in 0·25 ml of 2% HCl in ethanol) was diluted as required with isotonic saline within 30 min of use. The melphalan was administered i.p. at 0·5 ml/25 g body wt.

The nitroimidazoles used were:

(i) MISO: misonidazole; 1-(2-hydroxy-3-methoxypropyl)-2-nitroimidazole.
(ii) Ro 05-9963: desmethylmisonidazole; 1-(2,3-dihydroxypropyl)-2-nitroimidazole.
(iii) Ro 03-8800: 1-(2-hydroxy-3-morpholinopropyl)-2-nitroimidazole, hydrochloride.
(iv) Ro 03-8799: 1-(2-hydroxy-3-piperidinopropyl)-2-nitroimidazole, hydrochloride.
(v) RSU 1047: 1-(2-hydroxy-4-morpholinobutyl)-2-nitroimidazole.
(vi) METRO: metronidazole; 1-(2-hydroxyethyl)-2-methyl-5-nitroimidazole.

Compounds (i)–(iv) were supplied by Dr C. E. Smithen of Roche Products Ltd; compound (vi) by May and Baker Ltd, and compound (v) was synthesized by Dr I. Ahmed in this laboratory. Each nitroimidazole compound was dissolved in warm isotonic saline and, unless otherwise stated, administered i.p. at 0·5 ml/25 g body wt.

*Clonogenic assay.*—Tumour response was assayed by a modification of the soft-agar cloning technique described by Stephens *et al.* (1980). Eighteen hours after treatment, individual tumours were excised, scraped free of muscle, minced with curved scissors, weighed, suspended in 10 ml PBS, 10 mg trypsin and 0·5 mg DNase added, and rotated at 120 rev/min for 20 min in a 37°C chamber. A further 0·25 mg DNase was then added, the suspension filtered through 35$\mu$m-pore polyester mesh, centrifuged at 1000 rev/min for 5 min, resuspended in 10 ml Ham's F12 culture medium (supplemented with 15% donor calf serum, 60 $\mu$g/ml sodium benzyl penicillin, 100 $\mu$g/ml streptomycin sulphate, and 50 $\mu$g/ml neomycin sulphate) and refractile cells counted under a light microscope. Dilutions of the single-cell suspensions for measurement of their survival were based on counts of large cells only (*i.e.* $>12\ \mu$m diameter). The appropriately diluted cells, together with $10^4$ heavily irradiated feeder cells and $\sim 2{\cdot}5 \times 10^8$ washed August rat erythrocytes, were suspended in 1ml aliquots of 0·3% noble agar in Ham's F12 medium (supplemented with 20% donor calf serum and antibiotics as described above). Each aliquot was plated into a 30mm diameter plastic tissue-culture Petri dish containing a solidified layer of 1 ml of 0·5% noble agar in Ham's F12 medium (supplement as per aliquot). Nine replicate plates prepared from each tumour were incubated for 13–15 days at 37°C in a humidified atmosphere of 5% $O_2$, 5% $CO_2$ and 90% $N_2$. The resulting tumour colonies were counted using a low power microscope. Only compact colonies of $>50$ cells were counted. The plating efficiency (PE) of each tumour was calculated from the ratio of the number of colonies counted to the number of cells seeded.

### RESULTS

In the course of the present study 22 untreated tumours were used as controls. Because the tumours tended to grow into the body cavity accurate palpation proved difficult, and at excision the untreated control tumour weights varied from 240 to

FIG. 1.—The reduction in cell yield/g due to melphalan. Different symbols represent different experiments; the curve was fitted by eye.

450 mg. However, no dependence was observed between either cell yield/g and tumour weight or PE and tumour weight. This also holds true even if the range is extended from 180 to 570 mg. The mean number of cells harvested from the control tumours was $1 \cdot 1 \times 10^8$ cells/g (s.d. $2 \cdot 7 \times 10^7$), and their subsequent PE on incubation, 84% (s.d. 12%).

Relative to these control tumours, the cell yield/g was reduced after melphalan treatment, as shown by the scatter diagram in Fig. 1. This reduction in cell yield has been taken into account when expressing tumour survival. In the present studies, the survival has been expressed as surviving fraction/g tumour = relative PE × relative cell yield/g.

Fig. 2 shows the effect of single doses of melphalan (given 30 min after single doses of saline) on the survival of the MT tumour. The data, which show that obtained for all the melphalan controls done during the course of these experiments (as depicted by the different symbols), fit a survival curve computed by linear regression using least-squares fit analysis with N (extrapolation number) set at unity. Although the effect of melphalan was variable, it is evident that the scatter around the dose–response line occurred not from experiment to experiment, but rather from variations in individual tumour responsiveness. Consequently this dose–response line has been taken as the melphalan response for all experiments. It indicates that the MT tumour is sensitive to melphalan, a dose of 16 μmol/kg reducing survival by a decade.

To evaluate the effect of the nitroimidazoles on this melphalan activity, they were administered at various intervals either before, or after, the melphalan. Control experiments in which saline only was given up to 3 h before or after the melphalan showed no change in the activity of the drug; indeed, similar activity was observed when the saline dose was omitted. However, each of the 6

FIG. 2.—The dose response of melphalan activity against the MT tumour. Each datum point represents one tumour, different symbols representing different experiments.

FIG. 3.—Maximum cytotoxicity occurred when each of the 6 nitroimidazole compounds (at 2·5 mmol/kg i.p.) were given 0–30 min before melphalan (at 16 μmol/kg i.p.). Dashed lines: survival after melphalan only (derived from Fig. 2). Solid lines: survival after nitroimidazole given before or after melphalan (fitted by eye). Different symbols represent different experiments.

FIG. 4.—Dose-modifying potentiation of melphalan activity by each of the 6 nitroimidazoles (at 2·5 mmol/kg i.p.) given 30 min before. Different symbols represent different experiments, but for clarity the symbols for melphalan only activity (dashed lines) have been omitted as previously shown in Fig. 2. The solid lines were fitted by linear regression with N set at geometric mean of nitroimidazole alone toxicity. DMFs from slope ratios are shown.

nitroimidazoles, when given in combination with melphalan, reduced survival further. Maximum cytotoxicity occurred when the nitroimidazole was given 0–30 min before the melphalan (Fig. 3). To economize on the number of mice, full-time courses weie obtained only for MISO and 8799. With these compounds increased cytotoxicity still occurred when they were given up to 2 h before, or after, the melphalan.

Fig. 4 shows dose–response curves for the nitroimidazoles given at 2·5 mmol/kg 30 min before the melphalan (or saline in lieu). The data have been fitted to linear regression lines, with N set for each compound at the geometric mean survival after nitroimidazole treatment only. Although the values of N did not differ significantly between compounds (range: 0·78 for 1047 to 0·56 for 8799), the mean value for all compounds at 0·68 ± 0·03 (s.e. mean) does indicate that they did produce significant toxicity to ~30% of the cell population. The linear regression lines,

Fig. 5.—Potentiation of melphalan activity (16 μmol/kg) increased with nitroimidazole dose (given immediately before). Dashed line: melphalan only activity derived from Fig. 2. Solid line: fitted by eye through data points indicating no threshold dose. Different symbols represent different experiments.

originating from the individual values of N, indicate that the effect of the nitroimidazole in combination with melphalan was dose-modifying. The dose-modification factors (DMF) range from 1·6 for metronidazole to 2·2 for 8799.

Fig. 5 shows that although the potentiation of melphalan activity increases with misonidazole dose, the rate of increase falls. For the compound 8799 given at three-quarteers of the $LD_{50}$ dose, the maximum DMF is $\sim 3$. In all cases there was no evidence for a threshold dose since the response curves appear to extrapolate back to the survival fraction for the saline-plus-melphalan control (horizontal dotted line).

| Cmpd | MET | 9963 | MIS | 1047 | 8800 | 8799 |
|---|---|---|---|---|---|---|
| mg/g | 0·43 | 0·47 | 0·50 | 0·67 | 0·73 | 0·72 |
| mol.wt | 171 | 187 | 201 | 270 | 293 | 291 |
| $E^1_7$ | -486 | -389 | -389 | -375 | -368 | -346 |
| P | 0·96 | 0·11 | 0·43 | 0·34 | 0·37 | 8·5 |
| pKa | - | - | - | 6·65 | 6·15 | 8·71 |
| D | 0·96 | 0·11 | 0·43 | 0·29 | 0·35 | 0·40 |

Fig. 6.—Comparison of the potentiation by equimole doses of nitroimidazole given 30 min before melphalan (derived from Fig. 4). Full histograms represent ERs at $10^{-3}$ survival, smaller hatched histograms the DMFs from slope ratios; the difference reflects the nitroimidazole-only cytotoxicity. Potentiation may increase with increasing electron affinity ($E^1_7$); the distribution coefficients at pH 7·4 (D) are similar—as calculated from the pKa and partition coefficient measured of un-ionized form (P).

### DISCUSSION

The 6 nitroimidazoles investigated here are known to be effective hypoxic-cell radiosensitizers for the MT tumour (Sheldon & Hill, 1977; Adams *et al.*, 1982). They are also selectively toxic to hypoxic cells *in vitro* (Adams *et al.*, 1980; Stratford, personal communication). In the present work, the compounds when administered singly at 2·5 mmol/kg reduced MT tumour-cell survival *in situ* by about 30% (Fig. 4). This amount of cell kill is probably greater than might be expected on the basis of hypoxic-cell toxicity only. While the hypoxic fraction of the MT tumour has not been measured for the present implantation site, it is only 5% in sacral s.c. tumours (McNally & Sheldon, 1977) and 7% in thigh i.m. tumours (Stephens *et al.*, 1980). If the hypoxic fraction for the sacral i.m. tumours is similar in magnitude, then the 30% drop in cell-surviving fraction would imply some cytotoxic action against oxic cells also. Brown (1977) has reported that MISO alone was cytotoxic to 90% of EMT6 tumour cells, although only 30% were radiobiologically hypoxic.

Although the cytotoxic effects of the nitroimidazoles are significant, they are very small compared with the cytotoxic effect of melphalan alone. The large increase in cell kill observed for the combined treatment of nitroimidazole and melphalan compared with melphalan alone is clearly much greater than could be accounted for simply in terms of additivity of cell kill by each agent acting independently. The relative effectiveness of the nitroimidazoles as potentiators of melphalan are compared in Fig. 6. The administered doses of each compound were chosen to achieve equimolar levels (2·5 mmol/kg). The effectiveness of each compound is defined in Fig. 6 as both enhancement ratio (ER) and dose-modification factor (DMF). The ER is defined as the ratio of the melphalan doses required to give a surviving fraction of $10^{-3}$ and the DMF is the ratio of the linear slopes of the respective survival curves. The small difference between the values of ER and DMF for a given drug reflects the small but significant effect of the cytotoxic effect of the nitroimidazole alone.

The table of physical–chemical properties in Fig. 6 shows values of one-electron reduction potentials representative of relative electronaffinities ($E\ ^1_7$), octanol–water partition coefficients (P) and, where relevant, the pKa for the compounds. The 3 compounds 1047, 8800 and 8799 have a basic function in their side chains and this is reflected in their pKa values. However, all except 8799 are essentially un-ionized at biological pH. Hence for all except 8799 the measured coefficients of the compounds in their un-ionized form (P) are similar to their calculated distribution coefficients at pH 7·4 (D). Nevertheless, the values of D for all compounds are similar and no conclusions can be drawn from these data in regard to the influence of D on effectiveness of chemosensitization. However, data for other compounds without pKa considerations and covering a substantially greater range of partition coefficient (Sheldon & Batten, 1982; Workman & Twentyman, 1982) indicate that effectiveness can increase with increasing lipophilicity.

Although it is known that hypoxic cytotoxicity of nitroimidazoles *in vitro* increases with electron affinity (Adams *et al.*, 1980), it is not known what influence electron affinity has on the effectiveness of a nitroimidazole to potentiate the activity of chemotherapeutic agents. However, our data in Fig. 6 do show that from left to right the overall trend is for the enhancement ratios to increase, and this does correspond to an increase in electron affinity of the compounds.

It has been reported previously that maximum potentiation of melphalan activity towards the Lewis lung carcinoma occurred when MISO was given immediately before treatment with melphalan, although some potentiation occurred when given up to 2 h before or after (Rose *et al.*, 1980). In the MT tumour the timing appeared less critical, full potentiation occurring for all 6 of the nitroimidazoles when given 0–30 min before the melphalan (Fig. 3). For MISO and 8799 at least, some potentiation was still evident when the nitroimidazole was given up to 2 h before or after the melphalan.

Consideration of the mechanism of this potentiation will be dealt with in a subsequent paper. Since greater potentiation of melphalan activity has been reported in tumours than dose-limiting normal tissues (Rose *et al.*, 1980; Clement *et al.*, 1980), a therapeutic gain may be anticipated clinically. Although the nitroimidazole dose mainly used here (2·5 mmol/kg) is approximately 3-fold that clinically acceptable for MISO as a radiosensitizer (Dische *et al.*, 1977), the absence of a threshold dose (Fig. 5) suggests that some potentiation may occur at clinically acceptable doses. Indeed, as the magnitude of this effect has been reported to increase with prolonged contact time (Brown & Hirst, 1982), the effect would be expected to be greater clinically than here because of the shorter half-lives of these agents in mice than in man.

In conclusion, the present study has shown that the activity of the alkylating agent melphalan against the anaplastic MT tumour can be potentiated by nitroimidazole compounds in a dose-modifying manner. MISO, radiobiologically the most commonly used nitroimidazole, was not the most effective potentiator of melphalan activity; of the 6 compounds investigated in this paper, the most effective compound was Ro 03-8799.

We wish to thank: the Gray Laboratory of the Cancer Research Campaign for the donation of the MT tumour and WHT/GyfBSVS breeding nucleus; Mr J. Wallace and colleagues for establishing from this nucleus the Institute's own breeding colony; Dr T. Stephens and Mr J. Peacock for assistance in establishing the assay used; Mr J. Currant for manufacture of the incubator mixed gassing equipment; Drs I. J. Stratford and I. Ahmed of our department for helpful discussion and supply of RSU 1047 respectively; and the MRC for financial support.

### REFERENCES

ADAMS, G. E., SHELDON, P. W. & STRATFORD, I. J. (1982) How do we find better radiosensitizers? In *Progress in Radio-oncology* II (Eds Karcher *et al.*). New York: Raven Press, p. 275.

ADAMS, G. E., STRATFORD, I. J., WALLACE, R. G., WARDMAN, P. & WATTS, M. E. (1980) Toxicity of nitro compounds toward hypoxic mammalian cells: Dependence upon reduction potential. *J. Natl.Cancer Inst.*, **64**, 555.

BROWN, J. M. (1977) Cytotoxic effects of the hypoxic cell radiosensitizer Ro 07-0582 to tumor cells *in vivo*. *Radiat. Res.*, **72**, 469.

BROWN, J. M. & HIRST, D. G. (1982) Effect of clinical levels of misonidazole on the response of tumour and normal tissues in the mouse to alkylating agents. *Br. J. Cancer*, **45**, 700.

CLEMENT, J. J., GORMAN, M. S., WODINSKY, I., CATANE, R. & JOHNSON, R. K. (1980) Enhancement of antitumour activity of alkylating agents by the radiation sensitizer misonidazole. *Cancer Res.*, **40**, 4165.

DISCHE, S., SAUNDERS, M. I., LEE, M. E., ADAMS, G. E. & FLOCKHART, I. R. (1977) Clinical testing of the radiosensitizer Ro 07-0582—experience with multiple doses. *Br. J. Cancer*, **35**, 567.

HALL, E. J. & ROIZIN-TOWLE, L. (1975) Hypoxic sensitizers: Radiobiological studies at the cellular level. *Radiology*, **117**, 453.

HILL, R. P. & STANLEY, J. A. (1975) The response of hypoxic B16 melanoma cells *in vitro*, treatment with chemotherapeutic agents. *Cancer Res.*, **35**, 1147.

LAW, M. P., HIRST, D. G. & BROWN, J. M. (1981) The enhancing effect of misonidazole on the response of the RIF-1 tumour to cyclophosphamide. *Br. J. Cancer*, **44**, 208.

MARTIN, W. M. C., MCNALLY, N. J. & DE RONDE, J. (1981) Enhancement of the effect of cytotoxic drugs by radiosensitizers. *Br. J. Cancer*, **43**, 756.

MCNALLY, N. J. & SHELDON, P. W. (1977) The effect of radiation on tumour growth delay, cell survival and mouse cure using the same tumour system. *Br. J. Radiol.*, **50**, 321.

MULCAHY, R. T., SIEMANN, D. W. & SUTHERLAND, R. M. (1981) *In vivo* response of KHT sarcomas to combination chemotherapy with radiosensitizers and BCNU. *Br. J. Cancer*, **43**, 93.

ROSE, C. M., MILLAR, J. L., PEACOCK, J. H., PHELPS, T. A. & STEPHENS, T. C. (1980) Differential enhancement of melphalan cytotoxicity in tumour and normal tissue by misonidazole. In *Radiation Sensitizers* (Ed. Brady). New York: Masson Publishers. p. 250.

SHELDON, P. W. & BATTEN, E. L. (1982) Potentiation *in vivo* of melphalan activity by nitroimidazole compounds. *Int. J. Radiat. Oncol. Biol. Phys.*, **8**, 635.

SHELDON, P. W. & HILL, S. A. (1977) Hypoxic-cell radiosensitizers and tumour control by X-ray of a transplanted tumour in mice. *Br. J. Cancer*, **35**, 795.

SIEMANN, D. W. (1981) *In vivo* combination of misonidazole and the chemotherapeutic agent CCNU. *Br. J. Cancer*, **43**, 367.

SMITH, E., STRATFORD, I. J. & ADAMS, G. E. (1979) The resistance of hypoxic mammalian cells to chemotherapeutic agents. *Br. J. Cancer*, **40**, 316.

STEPHENS, T. C., COURTENAY, V. D., MILLS, J., PEACOCK, J. H., ROSE, C. M. & SPOONER, D. (1981) Enhanced cell killing in Lewis lung carcinoma and a human pancreatic carcinoma xenograft by the combination of cytotoxic drugs and misonidazole. *Br. J. Cancer*, **43**, 451.

STEPHENS, T. C., PEACOCK, J. H. & SHELDON, P. W. (1980) Influence of *in vitro* assay conditions on the assessment of radiobiological parameters of the MT tumour. *Br. J. Radiol.*, **53**, 1182.

STRATFORD, I. J., ADAMS, G. E., HORSMAN, M. R. & 4 others (1980) The interaction of misonidazole with radiation, chemotherapeutic agents, or heat. *Cancer Clin. Trials*, **3**, 231.

SUTHERLAND, R. M. (1974) Selective chemotherapy of non-cycling cells in an *in vitro* tumour model. *Cancer Res.*, **34**, 3501.

SUTHERLAND, R. M., EDDY, H. A., BAREHAM, B., REICH, K. & VANANTWERP, D. (1979) Resistance to adriamycin in multicellular spheroids. *Int. J. Radiat. Oncol. Biol. Phys.*, **5**, 1225.

TANNOCK, I. F. (1980) *In vivo* interaction of anticancer drugs with misonidazole or metronidazole: methotrexate, 5-fluorouracil and adriamycin. *Br. J. Cancer*, **42**, 861.

TWENTYMAN, P. R. (1981) Modification of tumour and host response to cyclophosphamide by misonidazole and by WR 2721. *Br. J. Cancer*, **43**, 745.

WORKMAN, P. & TWENTYMAN, P. R. (1982) Enhancement by electron-affinic agents of the therapeutic effects of cytotoxic agents against the KHT tumour: Structure activity relationships. *Int. J. Radiat. Oncol. Biol. Phys.*, **8**, 623.